# Development of a benchmarking toolkit for adolescent and young adult rheumatology services (BeTAR)

**DOI:** 10.1186/s12969-019-0323-8

**Published:** 2019-05-21

**Authors:** Ran A. Cai, Hema Chaplin, Polly Livermore, Martin Lee, Debajit Sen, Lucy R. Wedderburn, Nick Wilkinson, Rachel Jeffery, Andrea Kempa, Imogen Norton, Rachel Tattersall, Yiannis Ioannou, Despina Eleftheriou

**Affiliations:** 1 Arthritis Research UK Centre for Adolescent Rheumatology, University College London, London, UK; 2 Health Psychology Section, Psychology Department, Institute of Psychiatry, Psychology and Neuroscience, King’s College London, London, UK; 3 Paediatric Rheumatology Department, Great Ormond Street Hospital For Children NHS Foundation Trust, London, UK; 4 UCL GOS Institute of Child Health, University College London, London, UK; 5 Rheumatology Department, Newcastle Upon Tyne Hospitals, Newcastle, UK; 6 Rheumatology, University College London Hospitals, London, UK; 7 NIHR Biomedical Research Centre, Great Ormond Street Hospital For Children NHS Foundation Trust, London, UK; 8 Paediatric Rheumatology Department, Evelina London Children’s Hospital, Guys and St Thomas’ NHS Foundation Trust, London, UK; 9 Rheumatology, Northampton General Hospital NHS Trust, Northampton, UK; 10 Rheumatology Department, Royal Hallamshire Hospital, Sheffield, UK and Sheffield Children’s Hospital, Sheffield, UK

**Keywords:** Adolescent rheumatology, Standards of care, Benchmarking, Quality improvement, Healthcare services, Patient involvement, Toolkit

## Abstract

**Background:**

Young people (YP; 12–24 years old) with rheumatic diseases face many challenges associated with chronic illness in addition to the physiological and psychosocial changes of adolescence. Timely access to developmentally appropriate multidisciplinary care is key to successfully managing rheumatic diseases, but gaps in the care of this vulnerable age group still exist. This study aimed to develop a benchmarking toolkit to enable comparative evaluation of YP rheumatology services in order to promote best practice and reduce variations in service delivery.

**Methods:**

A staged and consultative method was used across a broad group of stakeholders in the UK (YP, parents/other carers, and healthcare professionals, HCPs) to develop this toolkit, with reference to pre-existing standards of YP-friendly healthcare. Eighty-seven YP (median age 19 years, range 12–24 years) and 26 rheumatology HCPs with 1–34 years of experience caring for YP have participated.

**Results:**

Thirty quality criteria were identified, which were grouped into four main domains: assessment and treatment, information and involvement, accessibility and environment, and continuity of care. Two toolkit versions, one to be completed by HCPs and one to be completed by patients, were developed. These were further refined by relevant groups and face validity was confirmed.

**Conclusions:**

A toolkit has been developed to systematically evaluate and benchmark YP rheumatology services, which is key in setting standards of care, identifying targets for improvement and facilitating research. Engagement from YP, clinical teams, and commissioners with this tool should facilitate investigation of variability in levels of care and drive quality improvement.

**Electronic supplementary material:**

The online version of this article (10.1186/s12969-019-0323-8) contains supplementary material, which is available to authorized users.

## Background

Advances in the field of paediatric and adolescent rheumatology over the past decade have decreased long-term morbidity and mortality rates [1]. This has resulted in a greater number of children with rheumatic diseases surviving into adulthood and having to negotiate transitions into adult services. As young people (YP) transfer from paediatric to adult rheumatology care, they need to develop an executive suite of skills including autonomy, resilience and self-management [2, 3]. This occurs in parallel to the immense physiological and psychosocial developmental changes that challenge all YP [4, 5]. Better organised services are therefore required to address the specific needs of this population.

Appropriately tailored interventions and healthcare provision remain central to minimizing the adverse impact of rheumatic diseases on physical and visual functions, psychosocial adjustments, general quality of life, as well as educational attainments during this vulnerable time. Failing to meet the needs of YP and families may negatively impact YP’s health and lead to disengagement with healthcare services [2, 6–8]. Disappointingly, unmet needs of YP with rheumatic diseases and gaps in care, particularly at transfer to adult healthcare provision, are still reported worldwide [9–11]. This is despite published guidelines for how to provide YP-friendly services [12–18] and the solid evidence base supporting the positive outcomes of planned and individualised developmentally appropriate care for YP [2, 6–8].

A number of previous recommendations outlined quality standards and performance measures that are specifically relevant to YP with juvenile-onset rheumatic diseases have either focused on transitional care [19, 20] or on specialised medical care guidelines for juvenile idiopathic arthritis (JIA) [21–24]. Efforts to measure care quality have also resulted in the development of generic self-assessment tools to evaluate standards of care for YP, two of which are endorsed by key professional bodies in the UK: 1) the You’re Welcome (YW) self-review tool that is applicable to various healthcare settings [25] and 2) the condition-specific tool for measuring Standards of Care (SOC) for Children and Young People with JIA [26]. Both YW and SOC tools provide a set of statements/questions to help service providers evaluate how well they are meeting various domains of YP-friendly care.

However, the majority of these previously published guidelines are descriptive rather than providing a quantitative assessment to allow for comparative service evaluations. In addition, previously developed tools may not be applicable to all YP living with different types of chronic inflammatory rheumatic diseases [21, 25]. It is thus essential to develop a benchmarking toolkit that can evaluate adolescent and young adult rheumatology services in order to promote best practice and reduce current variations in service delivery for this age group. The aim of this project was therefore to develop a comprehensive benchmarking toolkit for adolescent and young adult rheumatology services (BeTAR) that is applicable across both paediatric and adult services. BeTAR was developed in partnership with YP and under the auspices of the Barbara Ansell National Network of Adolescent Rheumatology, a network of adult and paediatric healthcare professionals (HCPs) caring for YP with rheumatic diseases across the UK. This toolkit will first enable evaluations of current clinical practice of YP rheumatology services in the UK before implementing it internationally.

## Participants and methods

**Fig. 1:**
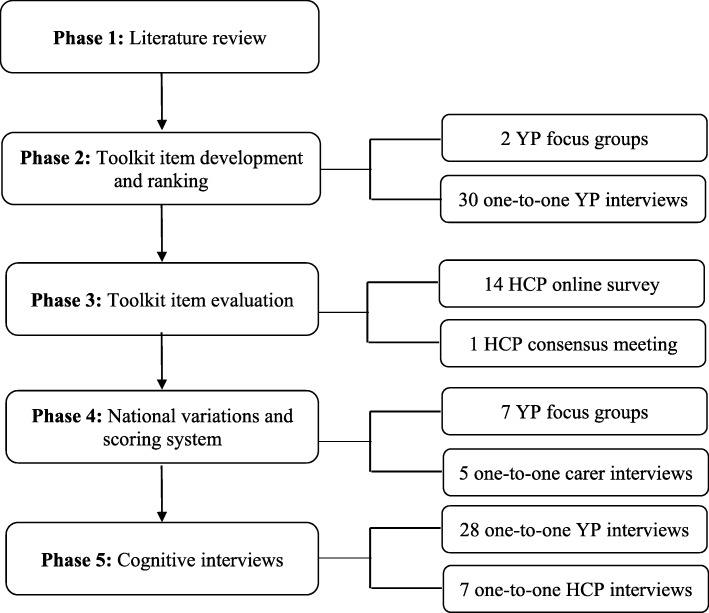
Study phases for developing BeTAR

BeTAR was developed and refined using an iterative process over five phases by combining findings from previous literature with results from focus groups (FGs), semi-structured interviews, and surveys from YP, parents/other carers, and HCPs (Fig. 1). A maximum variation sampling method was used to recruit participants to account for a wide range of backgrounds and experiences. We aimed to maximise engagement by offering different ways of data collection through both face-to-face and online methods. All participants could take part in multiple phases of the study. The study was granted ethics approval by the Office for Research Ethics Committees Northern Ireland (REC15/NI/0207) and informed consent and assent were obtained. The study was registered on the National Institute of Health Research portfolio of non-commercial studies (study ID: 19980).

### Phase 1: Literature review to define standards of care for rheumatology YP-friendly services

A systematic literature search was performed to establish any published standards or assessments for YP-friendly rheumatology services. All publications until June 2018 from four popular databases (PubMed, CINAHL, EMBase, and Web of Science) were searched. Initial search terms were obtained from MeSH (Medical Subject Heading) nomenclature and then mapped to appropriate database-specific terminology from selected publications (Table 1). Papers matching any combination of terms from the four concept areas in title, subject, keyword, and full text were retrieved for review. In addition, we searched websites of respected professional organisations for published guidelines and recommendations for YP’s healthcare to screen for additional standards. These included the following: Arthritis and Musculoskeletal Alliance (ARMA), British Society for Rheumatology (BSR/BSPAR), European League Against Rheumatism (EULAR), Paediatric Rheumatology European Society (PReS), American College of Rheumatology (ACR), Royal Australian College of General Practitioners (RACGP), Canadian Rheumatology Association (CRA), National Institute for Health and Care Excellence (NICE) and Department of Health and Social Care (DHSC).

**Table 1:** Literature search strategy

	Concept area keywords	Search	PubMed	EMBase	Web of Science	Professional organisations
A	“young adult*”, adolescent*, youth, teen*	A				475
B	transition*, service*, healthcare, “health care”, care	[A and B and C and D]	3586^a^	2349^a^	480	
C	quality*, model*, indicator*, standard*, tool*, evaluation*, benchmark*, criteria, guideline*, assessment*, measure*, recommendation*, performance*					
D	arthritis, “lupus erythematosus”, scleroderma, vasculitis, dermatomyositis, dermatopolymyositis, polymyositis)					
	Retrieved for review process		67	80	192	11

^a^ Top 500 hits (sorted by relevance) reviewed for inclusion

**Fig. 2:**
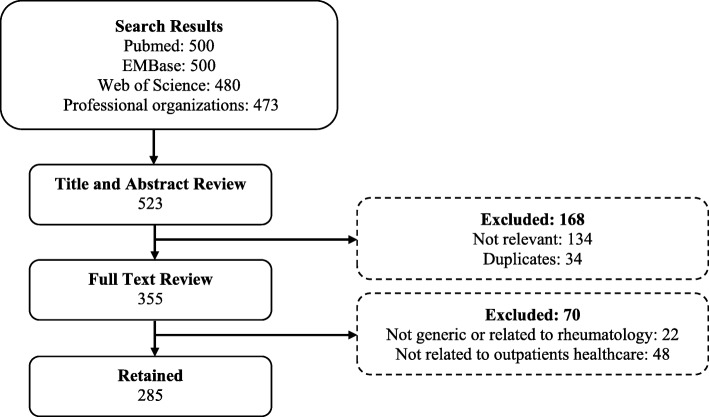
Literature search and review process

Studies looking at quality of care for YP-friendly rheumatology services written in English were identified (Fig. 2). Studies were excluded if they were not generic and focused specifically on chronic conditions that are not related to rheumatology. From these data, quality standards for YP-friendly services that are relevant for rheumatology care were reviewed by the core study team (RAC, DE, RT and YI). This helped define themes relating to what constitutes good healthcare provision for YP.

### Phase 2: Toolkit item development and ranking in partnership with adolescents and young adults

**Fig. 3:**
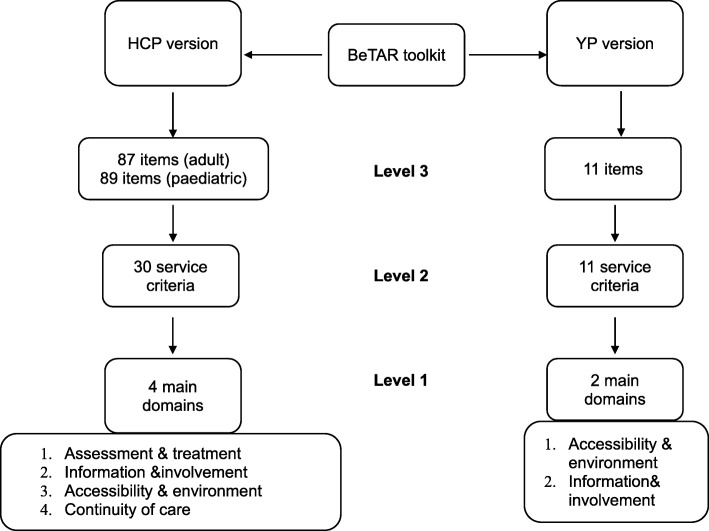
BeTAR structure

YP (12–24 years old) with chronic inflammatory rheumatic diseases cared for at a pediatric, adolescent and young adult rheumatology hospital unit in London were approached to take part in the study for a mixture of FGs and one-to-one interviews. All sessions were conducted, taped, and transcribed by the same researcher (RAC), who was not involved in participants’ clinical care. YP’s thoughts on these previously published standards of care were explored. They were asked what criteria need to be met by a service to provide the best possible care, and whether they felt any new standards or areas of care needed to be considered. Lastly, YP were asked to generate specific questions and response items for each criterion. As a result of this process, the toolkit developed comprised of two versions, one to be completed by HCPs and one to be completed by YP (Fig. 3). This procedure was repeated until saturation was reached and no new information and further criteria were suggested from YP [27]. The structure of the FGs and in-depth interviews are shown in Additional file 1. YP were then asked to prioritise all criteria based on their order of importance using a diamond ranking exercise, and each criterion was assigned a rank-order based on their ranked position [28]. YP also rated each criteria on a 5-point scale (1 = not important at all, 5 = very important).

### Phase 3: Toolkit item evaluation by HCPs

A multidisciplinary group of rheumatology HCPs reviewed the standards and rank-order assigned by YP. The process was completed through online surveys, followed by a face to face consensus meeting. Key areas covered were comprehensiveness, clarity, relevance, and feasibility of use for the proposed HCP toolkit. If ≥80% of HCPs endorsed a proposed standard or a way of combining multiple criteria, then this standard was included in the toolkit. If a criterion reached < 80% consensus it was discussed in the next phase with YP. These thresholds were also used to evaluate the question and response items, as well as the rank-order of each criterion. Lastly, HCPs were asked about the challenges of using the toolkit in practice.

### Phase 4: Accounting for multi-site variation and proposing a scoring system defined by YP

Phase 4 aimed to refine the toolkit by accounting for any multi-site variation, and to develop a quantitative scoring system. YP and their parents/carers were recruited from rheumatology outpatient’s clinics in Sheffield, Newcastle, Northampton, and London to participate in either FGs or one-to-one interviews. We chose these places to maximize variations in participants’ experiences by involving health services from larger, more ethnically diverse cities where less than 60% of the population are from the White ethnic group (London), as well as from smaller, less diverse cities with a more than 80% (Newcastle and Northampton) and 90% (Sheffield) of the population from the White ethnic group.

**Fig. 4:**
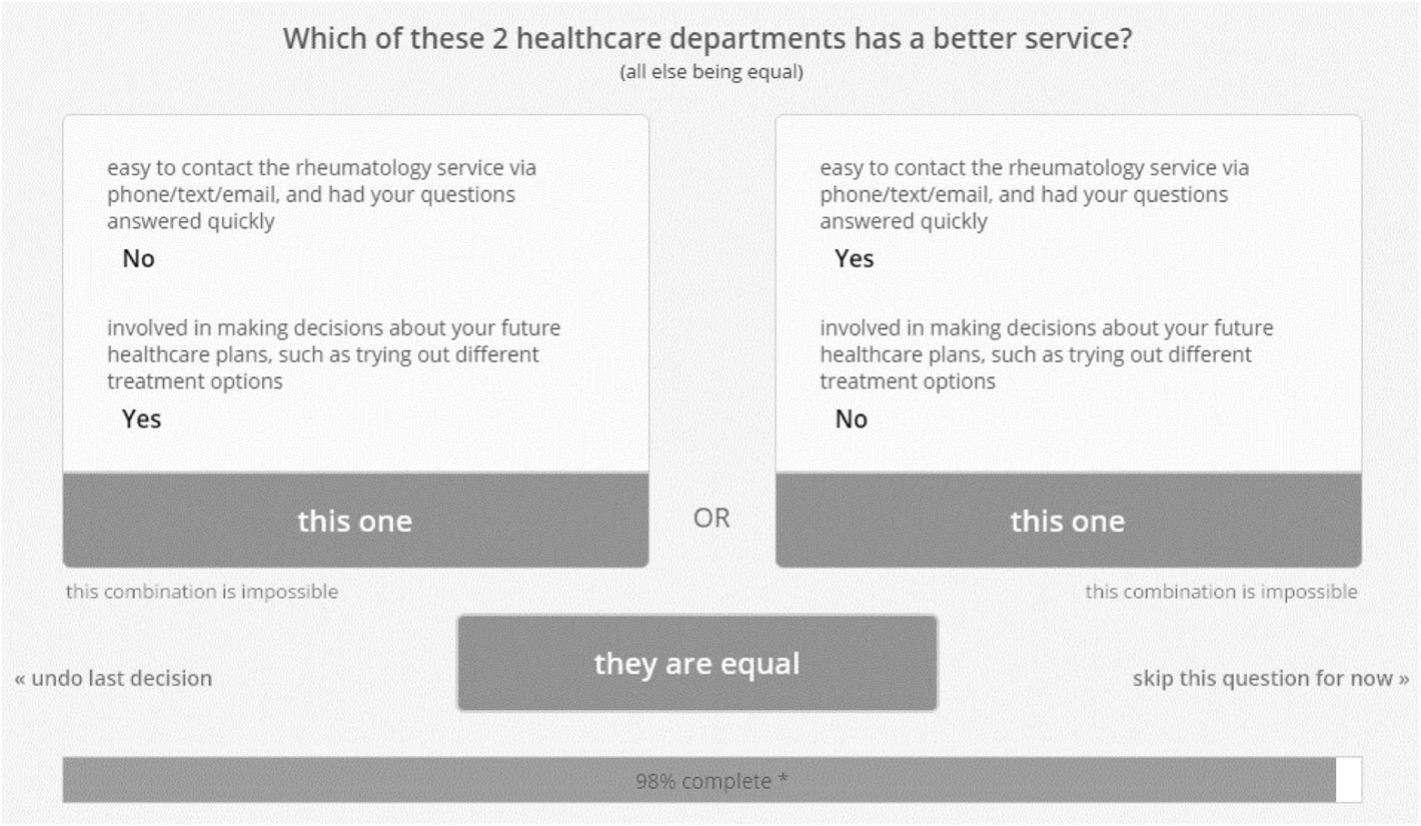
Example of a pairwise-ranking question from the 1000Minds conjoint-analysis survey

The 1000minds software [29], which is a decision-making program using the Potentially All Pairwise RanKings of all possible Alternatives (PAPRIKA) method [30], was used to develop the scoring system for both versions of the toolkit. Participants were first asked to review the toolkit’s content and were then given repeated comparisons between two service criteria through 1000minds. They were asked to choose which of the two service criteria is more important or whether they are equally important (Fig. 4). Each criterion received a ‘preference value’ according to the PAPRIKA method; criteria with greater importance received higher preference values and were thus assigned more points than criteria with lower preference values.

During this process, the rank-order of each criterion proposed by YP in phase 2 and by HCPs in phase 3 were also discussed to encourage YP to reassess their judgements in light of the comments and feedback provided by other participants [31]. Relative weights of the criteria were used to develop a scoring system such that when all item responses are considered together, the maximum score possible for each version of the toolkit is 100 and the minimum score is 0.

### Phase 5: Cognitive interviews to evaluate face validity and usability

Phase 5 aimed to evaluate face validity and test the usability of the toolkit through individual cognitive interviews (CI). Sample size used for cognitive interviews are normally small, and five participants can be sufficient [32–35]. Purposeful sampling [36] was used to ensure HCPs from various rheumatology centres and YP with diverse disease characteristics and demographics were included. Participants were interviewed in person or over the telephone. A mixture of think-aloud and verbal probing techniques were used in order to gain maximal information and to encourage participants to talk about any words or concepts that they found troublesome (see Additional file 2) [34, 35]. For any item that participants judged as ‘medium’ or ‘hard’ to understand, they were asked to suggest ways to reword the item to improve comprehension. The data gathered were used to modify and improve question items and response choices.

### Data analysis

Qualitative responses from FGs and one-to-one interviews were taped, transcribed, and analysed using content analysis to identify key themes and categories [37]. To reduce the chance of bias, transcripts were reviewed by two researchers (RAC and DE) to identify major themes [38]. Differences were resolved by discussions with two more researchers (YI and HC) until consensus was reached. Data analysis was carried out using NVivo software [39] for coding data with similar contents into meaningful categories and overarching themes. In addition, content analysis was performed according to centre and age group (12–15 years and 16–24 years).

## Results

### Participants

Out of 95 YP who were approached, eight did not participate either because they did not have time (*n* = 3) or were not interested (*n* = 5). A total of 87 YP (21 males) aged 12–24 with rheumatic diseases and five parents/carers of YP participated in the study (Table 2). The median age of diagnosis was 10.5 (range 1–16) and median disease duration was 9 years (range 1–22). Out of 29 rheumatology HCPs who were approached for the study, 26 agreed to take part. The median number of experience in adolescent rheumatology was 11 years (range 1–34).

**Table 2:** Demographic characteristics of YP and HCPs included in the study

	Phase 2	Phase 3	Phase 4	Phase 5
Total no. of YP^a^, *n*	38		27	28
Age				
Median in years (range)	19 (15–24)		18 (12–24)	19 (12–24)
Mean in years (SD)	19.1 (2.7)		18.5 (3)	18.8 (3.1)
Age of diagnosis				
Median in years (range)	10 (1–16)		10 (1–16)	10.5 (2–15)
Mean in years (SD)	9.5 (4.4)		9.4 (4.8)	10 (3.8)
Sex				
Female, *n* (%)	31 (82)		18 (67)	17 (61)
Male, *n* (%)	7 (18)		9 (33)	11 (39)
Ethnicity				
White, *n* (%)	24 (63)		17 (63)	18 (64)
Asian, *n* (%)	9 (24)		5 (19)	7 (25)
Black, *n* (%)	3 (8)		3 (11)	3 (7)
Other, *n* (%)	2 (5)		2 (7)	2 (4)
Type of diagnosis				
JIA, *n* (%)	27 (71)		20 (74)	17 (61)
JSLE, *n* (%)	7 (18)		4 (15)	4 (14)
JDM, *n* (%)	4 (11)		3 (11)	7 (25)
Total no. of HCPs^b^, *n*		22		7
Clinician, *n* (%)		16 (73)		5 (71)
Clinical nurse specialists, *n* (%)		5 (23)		2 (29)
Physiotherapist, *n* (%)		1 (4)		0
Years in practice				
Median (range)		10.5 (1–34)		11 (3–34)
Mean (SD)		13.3 (9.3)		14.5 (8.9)

^a^ Six YP took part in multiple phases of the study

^b^ Three HCPs took part in multiple phases of the study

### Phase 1: Summary of literature review defining standards of optimal care for rheumatology YP-friendly services

The literature search identified 1953 articles; after screening for full text and duplicates, 285 articles remained. In total, 48 distinct criteria pertaining to quality of healthcare services for YP in rheumatology services were extracted from these articles and were grouped into 6 themes: provision of information or education, preparation for transition to adulthood, staff expertise/support, YP involvement, service efficiency, and service accessibility. All these themes were discussed with YP in the following phases.

### Phase 2: Developing and ranking items based on FGs and interviews with YP

Two FGs (*n*_*1*_ *=* 5, *n*_*2*_ *=* 3) and 30 one-to-one interviews were conducted with YP. During this phase, YP suggested 17 new criteria that included: providing specific pain-management information; self-injection of methotrexate taught by a rheumatology nurse; and sharing and explaining test/assessment results (e.g., blood tests). All 17 of these new criteria proposed by YP, as well as all the criteria and standards derived from previous literature, are listed in Additional file 3. The five most important criteria according to YP were: HCPs’ expertise/knowledge in adolescent rheumatology, accessing effective therapies and treatments; timely access to treatments; monitoring symptoms, and accessing urgent consultations. All 65 criteria were included in the preliminary version of the toolkit. YP also generated a list of questions and response items to define and assess each criterion. Moreover, YP suggested a YP version of the toolkit to assess the quality of care from the patient’s perspective.

### Phase 3: Further toolkit refinement and evaluation by HCPs

Fourteen HCPs participated in an online survey and eight HCPs attended the consensus meeting. The group agreed on 18 of the original criteria to be included in the toolkit and 42 original criteria were combined into 13 new criteria. For example, providing out-of-hour appointments and phone/skype consultations were combined into one inclusive criterion for “convenient appointments”. Five criteria were excluded as they were ranked low in importance by YP and > 80% of the HCPs voted in favour of excluding them. These were: providing information on nutrition, providing information on alcohol and drugs, providing information on sexual health, accessibility for patients with physical disability, and easy access to the hospital by public transport. HCPs also suggested higher rank-orders for developmentally appropriate care (e.g., providing transitional care plan) and for providing easy ways to contact the service with timely responses, which were ranked relatively low by YP.

**Fig. 5:**
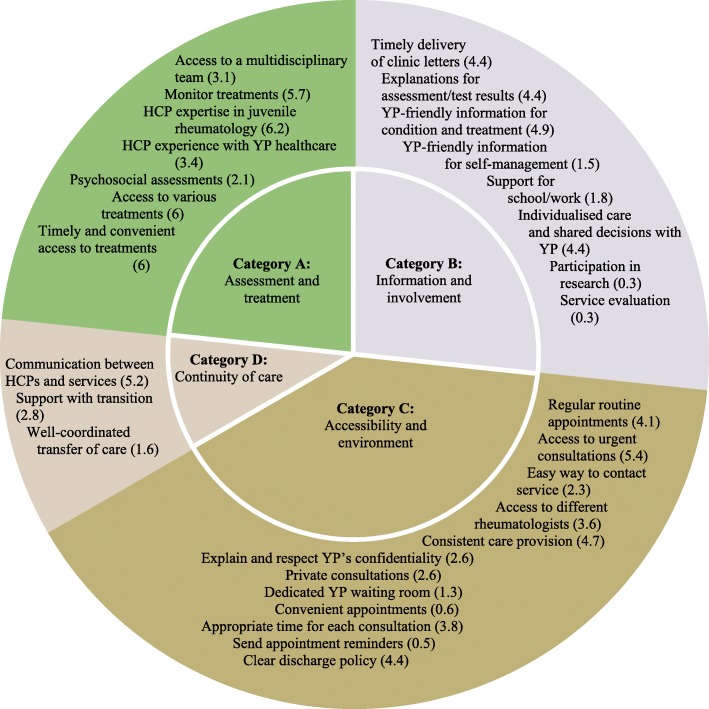
Categories and criteria for the HCP version; preference values are presented in parentheses

Moreover, HCPs agreed to remove 18 out of the 105 question items that are likely to generate the same responses from all services, and hence are not discriminatory. These include whether or not services provide blood monitoring for anti-inflammatory treatments and whether or not services send out clinic letters to patients. Forty-seven items were kept without revision and 40 items were either clarified to improve understanding or reworded to better identify challenges and issues with care provision. HCPs also discussed differences between adult and adolescent/paediatric care, and how the final item pool needs to account for these variations. As a result, two additional response items were added for services seeing YP under the age of 16, which are whether services provide educational information and resources to parents/carers and whether service policies (e.g., discharge policy) are explained to parents/carers. The revised toolkit at this stage contained 30 criteria with 89 question items for paediatric services seeing YP under 16 years old, and 87 response items for services seeing YP who are 16 years old or older (Fig. 5). Out of these 30 criteria, YP selected 11 criteria to be included in the YP version of the toolkit (see Additional file 4). These modifications were discussed with YP during phase 4.

Lastly, HCPs agreed with the need for a YP facing version of the toolkit in order to address problems with care identified from both patients’ and providers’ perspectives. Both versions have similar structures with three levels of assessment. Level one includes overarching domains made up of individual criteria (level 2), which are assessed using different question items (level 3). The overarching domains from level 1 are: assessment and treatment, information and involvement, accessibility and environment, and continuity of care.

### Phase 4: Developing a weighted scoring system

Seven FGs with 27 YP (*n*_*1*_ *=* 5, *n*_*2*_ *=* 3, *n*_*3*_ *=* 3, *n*_*4*_ *=* 4*, n*_*5*_ *=* 4*, n*_*6*_ *=* 5*, n*_*7*_ *=* 3) from four cities across the UK and 5 one-to-one interviews with parents/carers of YP were conducted. Participants from this phase discussed the final criteria and question items, and agreed with almost all adaptations made by HCPs in phase 3. YP were also asked to complete the 1000minds programme collaboratively as a group during each FG. They were presented with an average of 156 pairwise-ranking for the HCP version, and an average of 78 pairwise-ranking for the YP version.

The mean preference values (derived using 1000minds) from all 7 FGs ranged from 0.3 to 6.2 for the HCP version in which 0.3 reflected the lowest and 6.2 the highest importance (see Additional file 5). For the YP version, the mean preference values ranged from 3.7 to 13.9 (see Additional file 4). Intra-class correlation estimates of these preference values based on mean-rating of the FGs (*k* = 7), absolute agreement, and 2-way random-effects model were high (0.89), indicating good agreement between YP regarding the priority weighting given to these items. A preliminary scoring system based on the mean preference values was developed. This scoring approach across multiple domains allows for a good score in one domain to compensate for a poor score in another domain.

### Phase 5: Face validity and usability of the toolkit

#### YP version

Overall, CIs with YP (*n* = 28) showed that they were able to respond to the items without assistance and believed that the toolkit is easy to understand and feasible to complete (< 2 min) without compromising its comprehensiveness. YP suggested to replace the word “doctor” with “rheumatology team member” and also preferred a binary yes/no response format instead of a continuous scale. A “not relevant for my care” response option was added as well. In terms of the time frame, all YP suggested that answering the toolkit for their experiences every 6 months would be appropriate. Lastly, toolkit readability as measured using the Flesch-Kincaid Grade Level provided by Microsoft Word was 5.2, indicating that the toolkit is easily understood by YP aged 10 and above [40].

#### HCP version

CIs with HCPs (*n* = 7) demonstrated that the toolkit was completed with ease within a reasonable amount of time (15 min), and that it captured all essential quality indicators. HCPs suggested that it would be useful and feasible to complete the toolkit annually. Most participants demonstrated high understanding of the questions, were able to follow instructions appropriately, and could easily retrieve answers to each question. HCPs were asked to paraphrase certain question items in their own words and minor improvements were made by adapting terminologies that are commonly used by HCPs. In addition, an “other” option was added for multiple choice questions as well as an option to enter free-text responses.

## Discussion

This report describes a highly consensus-based methodology underpinning the development of BeTAR, a benchmarking toolkit for adolescent and young adult rheumatology services. The conceptual model of developmentally appropriate rheumatology services that emerged from previous work was further explored through discussions and interviews with stakeholders, which facilitated the identification of key quality measures to include in the toolkit. We worked in close collaboration with YP with chronic inflammatory rheumatic diseases in all phases of this study in order to confidently capture the multitude of service areas that truly reflect what YP want and need. Combining previous guidelines and recommendations for YP-care with new criteria derived by YP helped develop a list of items to characterize and assess service experience, and evaluate service provision in a comprehensive and YP-relevant way. Moreover, the YP version of the toolkit can encourage sustained engagement and involvement from YP, which is central to making real, constructive changes to the provision of care [41, 42].

Two additional key features of BeTAR are that it is widely applicable and that it can generate a quantitative score. First, instead of focusing only on transitional [19] or specialised medical care [21], BeTAR can be seen as one overarching toolkit that covers all aspects of rheumatology-specific needs for YP. The toolkit is also designed to be used across all rheumatology services that are seeing YP, regardless of whether they are in a paediatric or adult setting. Second, each criterion was assigned a weighted score based on their relative priorities for YP. The items in the current toolkit can therefore facilitate auditing and assessing performance levels, and to more easily identify gaps in performance, monitor progress, and realise opportunities for improvement in rheumatology services. Implementation of the proposed toolkit should thus facilitate investigations of variability between services and across networks, identify current levels of care and inspire future quality improvement programmes for YP with rheumatic diseases.

The application of BeTAR into clinical care will require a staged approach. The first step will be an initial data collection exercise evaluating adolescent and young adult rheumatology services across the UK and in the next phase internationally. This data collection phase will assess how well the standards are being met in terms of the quality, outcome, and experience of care for YP and how these relate to health outcomes. Describing and understanding current clinical practice through this process will enable services to target areas of poor performance and improve clinical care. We anticipate that the overall process can additionally facilitate effective quality assessment for service commissioning. For instance, in diabetes care there is evidence that investment in regional networks and the introduction of a Best Practice Tariff mandating participation in audit and benchmarking evaluation of services has resulted in improvements in outcome [43, 44]. This could also be the case for YP-friendly rheumatology services.

Even though we made an effort to establish the wider views of YP and HCPs by extending the exercise to several centres, we cannot exclude that responder bias may have influenced the views expressed. For example, we were only able to include clinical nurse specialists and physiotherapists in our sample, which may have limited the scope of the toolkit to represent the views of other allied HCPs such as psychologists and occupational therapists. In addition, although participation rate was high (92%) and we aimed to recruit participants from diverse ethnic backgrounds, translation services were not offered to participants; it is therefore possible that some families refused to participate due to a lack of command of English.

Moreover, ranking of criteria by YP could vary greatly between different healthcare systems and access to treatments from different parts of the world. It is possible that some of the items removed from the current toolkit for UK-based rheumatology services could be relevant in non-UK settings and may need to be considered further during the next steps of an international validation exercise. We also acknowledge that quality measures may change over time and therefore regular re-evaluation of the toolkit content is required. In addition, collecting data in itself is not adequate and the data must be utilized to actually deliver the change needed to drive quality improvements. This requires a concerted effort and commitment from both HCPs and YP, as well as input and involvement from healthcare managers. Thus our long-term goal is to establish a fit-for-purpose IT system, where the toolkit can be accessed electronically by service providers and HCP. This interactive portal (already under development) will not only allow for visual comparative evaluation against other hospital trusts, but will also refer to published resources and recommendations, foster information sharing and collaborative learning and thus allow individual centres to improve their services.

## Conclusions

In summary, through a multistage process involving several FGs, interviews, consensus meetings, and rating exercises, we developed a toolkit to benchmark and evaluate YP rheumatology services. Improved service delivery at local (trust), regional (clinical networks), UK wide, and international levels against these important criteria will likely identify potential areas for healthcare quality improvement, which is key to ensuring positive clinical outcomes for young people living with chronic rheumatic diseases.

## Additional files

Additional file 1: Toolkit development focus group guide. (DOCX 20 kb)

Additional file 2: Cognitive interview. (DOCX 17 kb)

Additional file 3: Summary of previously established standards and additional standards proposed by YP. (DOCX 17 kb)

Additional file 4: YP toolkit, with scores for each criterion presented in parentheses. These are derived from the average preference values from the 1000minds decision-making software. A higher preference value means a higher importance of that criterion for YP. (DOCX 16 kb)

Additional file 5: HCP toolkit. (DOCX 109 kb)

## Abbreviations

BeTAR: Benchmarking toolkit for adolescent rheumatology (a toolkit developed to measure service quality)
CI: Cognitive Interview
FG: Focus Group
HCP: Healthcare Provider
JIA: Juvenile Idiopathic Arthritis
PAPRIKA: All Pairwise RanKings of all possible Alternatives (a decision-making method for prioritising criteria)
SOC: Standards of Care
YP: Young People
YW: You’re Welcome (published quality criteria for young people friendly health services)
