# Supplementary material for: Development of a benchmarking toolkit for adolescent and young adult rheumatology services (BeTAR)
Source: Pediatr Rheumatol Online J. 2019 May 21;17:23. doi: 10.1186/s12969-019-0323-8 (PMC6528251; doi:10.1186/s12969-019-0323-8)
Supplement: Supplementary file 1 — Toolkit development focus group guide. (DOCX 20 kb) [file 12969_2019_323_MOESM1_ESM.docx]

Additional file 1: Toolkit development focus group guide

# Introductory questions

1. What do you like about the rheumatology services that are provided for young people?
2. What do you dislike about the rheumatology services that are provided for young people?
   1. Have your needs been met? If not then what was lacking?
   2. How can these issues be solved or improved?
   3. What do you consider an acceptable solution to these problems?
3. What criteria do you think is required for an excellent service and quality of care?
4. What types of support are important for you?

***Brainstorming:*** *Write down criteria generated by patients on sticky notes*

# Prioritizing exercise

***Diamond ranking:*** *Ask participant to arrange their own criteria (written on sticky notes) as well as the 25 criteria provided by the interviewer (printed on paper) in a diamond shape, ranking them by position where the most important criteria is at the top and the least important at the bottom. This will provide information on how the criteria are prioritized in relation to each other.*

*Participants will then be asked to score each criteria using a 5-point scale on their* ***importance*** *(1=not at all important, 2=slightly important, 3=moderately important, 4=very important, 5=extremely important).* ***Put the ratings on power-point so participants and look at them when ranking***

***Write down importance ratings on the card itself***

Thank you very much for your interest in a research study that we are running at UCLH. The project aims to develop a “toolkit” that can measure service qualities provided by rheumatology departments, and can be used to help improve healthcare services for young people in the UK.  We would therefore like to invite you to a Focus Group with other young people at UCLH, where we can listen to your comments and suggestions.

1. The project aims to develop a “toolkit” that can measure service qualities provided by rheumatology departments, and can be used to help improve healthcare services for young people in the UK.  The project aims to develop a “toolkit” that can measure service qualities provided by rheumatology departments, and can be used to help improve healthcare services for young people in the UK.  The project aims to develop a “toolkit” that can measure service qualities provided by rheumatology departments, and can be used to help improve healthcare services for young people in the UK.  Could you please arrange these criteria in order of importance for you, with the top being most important and highest priority, and bottom being of least priority.
2. WssPlease rate how important each criteria is on a scale of 1 – 5 as well.
3. Which of these criteria does a hospital need to have in order to be considered “excellent”, “good”, “adequate”, or “bad”?
4. What other criteria would you add to the list? Have we covered everything that is important?

# Follow-up questions

1. Why is this The project aims to develop a “toolkit” that can measure service qualities provided by rheumatology departments, and can be used to help improve healthcare services for young people in the UK.  important/not important or relevant for you?
2. Can you give me an example of when this worked/didn’t work?
3. Could you please clarify/define what you mean by this? (e.g., what is considered as convenient, fast, good, clear)
4. Have you ever used these services?

# Conclusion

**Concluding question**

- Of all the things we’ve discussed today, what would you say are the most important issues you would like to express about the toolkit development?
