# Supplementary material for: Development of a benchmarking toolkit for adolescent and young adult rheumatology services (BeTAR)
Source: Pediatr Rheumatol Online J. 2019 May 21;17:23. doi: 10.1186/s12969-019-0323-8 (PMC6528251; doi:10.1186/s12969-019-0323-8)
Supplement: Supplementary file 2 — Cognitive interview. (DOCX 17 kb) [file 12969_2019_323_MOESM2_ESM.docx]

Additional file 2: Cognitive interview

1. Are the introductions and summaries about the toolkit clear?
2. Can you rephrase what you think this question is asking?
3. Is the question ‘easy’, ‘medium’ or ‘hard’ to understand?
   1. How could we change the words to make it clearer?
4. How did you choose your answers for this question (i.e., what did you think of when answering this question)?
   1. Is it difficult to know what to answer for this question?
      1. How could we make it easier for you to answer?
      2. What do you think about the response choices?
   2. Do you need additional resources to answer this question? If so, what (e.g., audit data, patient-reported experiences)?
   3. Do you need to consult other people? If so, who?
   4. Can you provide evidence to support your answer or is it too subjective?
5. Are the response options appropriate for this question?
6. How much do you think it costs your service to fulfil this criteria (or the potential cost if not yet implemented)?
7. General questions:
   1. What period of time were you thinking about when you responded to these questions? (e.g., should we ask in the past year?)
   2. Please tell me what “average” means to you—how did you come up with the answer?
8. Ratings

|  | Strongly disagree |  |  |  | Strongly agree |
| --- | --- | --- | --- | --- | --- |
| 1. Overall it was easy to understand and complete this toolkit. | 0 | 1 | 2 | 3 | 4 |
| 1. I believe this toolkit can accurately assess service quality for young people rheumatology. | 0 | 1 | 2 | 3 | 4 |
| 1. This toolkit can help improve our services. | 0 | 1 | 2 | 3 | 4 |
| 1. Overall, I am satisfied with this toolkit. | 0 | 1 | 2 | 3 | 4 |
| 1. I found this toolkit very useful and appropriate to evaluate my service quality. | 0 | 1 | 2 | 3 | 4 |

1. Is the on-line version easy to read and complete?
   1. Should the text boxes be bigger?
   2. Should we provide a paper version as well?
2. Additional comments?
