# Supplementary material for: Development of a benchmarking toolkit for adolescent and young adult rheumatology services (BeTAR)
Source: Pediatr Rheumatol Online J. 2019 May 21;17:23. doi: 10.1186/s12969-019-0323-8 (PMC6528251; doi:10.1186/s12969-019-0323-8)
Supplement: Supplementary file 3 — Summary of previously established standards and additional standards proposed by YP. (DOCX 17 kb) [file 12969_2019_323_MOESM3_ESM.docx]

Additional file 3: Summary of previously established standards and additional standards proposed by YP.

| **Domains** | **Standards established from literature search** | **Additional items proposed by YP** |
| --- | --- | --- |
| 1. Information & education | - Information and resources tailored to YP’s (and carers’) unique needs.   - about YP specific rheumatic condition^a,b,1^   - about treatment administration and side effects^a,b,1^   - about managing symptoms^a,b,1^   - about emotional health^a,1^   - about financial issues^a,1^   - about employment and educational issues^a,1^   - Opportunities to meet similar peers^a,b,1^   - about nutrition and healthy eating^c^   - about alcohol and drugs^c^   - about sexual and reproductive health^c^ - Easily accessible information for YP (e.g., digital resources)^1^ - Enough face-to-face clinic time to ask questions^b^ | - Providing specific pain-management information^1^ |
| 1. Preparation for adulthood/ adult care | - Gradual transition process starting when YP are 11-13 years old^a,2^ - Information about transition to adult services and what YP can expect (e.g., differences in healthcare settings)^2^ - Preparing YP for adult responsibilities/independence in terms of self-management^2^ - Discuss with YP their long-term health needs^2^ - Collaborative links/pathways between paediatric and adult care providers^3^ - Continuity of care with no gaps/interruptions to established treatments^a,3^ - Comprehensive information transfer of patient history from paediatric to adult team^a,3^ - Transition coordinator/named key staff member acting as a bridge between paediatric and adult services^a,4^ | - Having an adult rheumatologist/nurse who can meet YP before transitioning into their care^4^ |
| 1. Staff expertise & support | - Provision of holistic/multidisciplinary care (CNS, ophthalmology, physiotherapy, occupational therapist, and clinical psychologist)^a,b,5^ - Appropriate referrals to specialists^a,b,5^ - Privacy during consultations^b,6^ - Good communication/shared care with local services and between professionals^a,b,7^ - Appropriate intervals between consultations/assessments^a,8^ - School and work-based support/advocacy^a^ - Staff experience working with YP (e.g., understanding YP’s needs, communicating using developmentally appropriate language)^a^ - Staff expertise/knowledge and experience in YP rheumatology - Appropriate monitoring of disease activity, uveitis, pain levels, and side-effects for DMARDs - Monitoring/assessing psychosocial wellbeing and general quality of life - adolescent rheumatology^b^ - Trust and confidentiality^b^ | - Referral to pain specialists^b,5^ - Assuring patients that all doctors are part of the same team with good communication between them^7^ - Accessing more frequent or longer consultations depending on health status/urgency^b,8^ - Being informed of who they will be seeing at their consultation^9^ - Ensuring all doctors are familiar with patients’ history and current situation^b,9^ - Self-injection of methotrexate taught by an appropriate rheumatology nurse - Able to request and see the same or a different rheumatologist - Sending appointment reminders - Being able to access different types of treatments and medications |
| 1. Patient involvement | - Providing personalised care/transition plans (e.g., allowing flexible “transition time” depending on YP’s readiness)^a,2^ - Allowing YP to decide who they want to attend consultations with (e.g., with or without carer)^6^ - Discussing treatment options with YP and listening to their opinions and needs^b,10^ - Involving YP throughout the treatment process and helping them make informed decisions about their treatments^a,b,10^ - Opportunity to be involved in clinical trials/studies - Providing YP-friendly feedback/complaints procedure and including YP’s views and feedback in service design and development | - Sharing and explaining test/assessment results (e.g., blood tests) in clinic letters^b^ |
| 1. Service efficiency | - Timely referrals^a,5^ - Sending clinic letters within a reasonable time frame^11^ - Timely response to queries^b,12^ - Quick and convenient access to treatments^a^ | - Addressing clinic letters to patients^11^ |
| 1. Service accessibility | - Access to rheumatology helplines^a,b,12^ - Out-of-hour appointments^a,13^ - Scheduling combined appointments with appropriate members of the MDT into one day^a,13^ - YP-friendly environment (e.g., reading materials, multi-media)^a^ - Easily accessible by public transport^c^ - Accessible by YP with physical disability or sensory impairments^c^ | - Contacting the service in multiple ways (e.g., texts, emails)^b,12^ - Ability to contact MDT services directly^b,12^ - providing phone/skype consultations^13^ - Having an appropriate policy of non-attendance that is clearly explained |

^a^Criteria generated by YP during unprompted information collection in phase 2

^b^Criteria included in YP toolkit

^c^Lowest rated criteria by YP that HCPs agreed to exclude

^1^**Combined criterion 1:** Providing educational information and resources

^2^**Combined criterion 2:** Individualised transition care plan

^3^**Combined criterion 3:** Having an agreed transition pathway with local centres

^4^**Combined criterion 4:** Having (a) key individual(s) at both centres during transition

^5^**Combined criterion 5:** Providing multidisciplinary care

^6^**Combined criterion 6:** Safe/comfortable consultation environment

^7^**Combined criterion 7:** Shared care and good communication among HCPs

^8^**Combined criterion 8:** Appropriate consultation intervals

^9^**Combined criterion 9:** Facilitate relationship continuity between HCPs and YP

^10^**Combeind criterion 10:** Shared decision-making with YP regarding their care

^11^**Combined criterion 11:** Receiving clinic letters

^12^**Combined criterion 12:** Easily contact rheumatology and MDT services

^13^**Combined criterion 13:** Convenient appointments
