# Supplementary material for: Development of a benchmarking toolkit for adolescent and young adult rheumatology services (BeTAR)
Source: Pediatr Rheumatol Online J. 2019 May 21;17:23. doi: 10.1186/s12969-019-0323-8 (PMC6528251; doi:10.1186/s12969-019-0323-8)
Supplement: Supplementary file 4 — YP toolkit, with scores for each criterion presented in parentheses. These are derived from the average preference values from the 1000minds decision-making software. A higher preference value means a higher importance of that criterion for YP. (DOCX 16 kb) [file 12969_2019_323_MOESM4_ESM.docx]

**Additional file 4:** YP toolkit, with scores for each criterion presented in parentheses. These are derived from the average preference values from the 1000minds decision-making software. A higher preference value means a higher importance of that criterion for YP.

|  | Yes | No | This is not relevant to me |
| --- | --- | --- | --- |
| 1. Were you given enough time with the doctor during your rheumatology consultations? (4.9) |  |  |  |
| 1. Were you able to get an earlier appointment with the rheumatology team if you had an urgent issue? (13.9) |  |  |  |
| 1. Was it easy for you to contact the rheumatology service via phone/text/email, and have your questions answered quickly? (10.5) |  |  |  |
| 1. Do you feel that everyone from the rheumatology team knows your medical history and knows how to meet your current healthcare needs? (9.4) |  |  |  |
| 1. Do you trust everyone from the rheumatology team, and feel that they respect your confidentiality and privacy (e.g., you can seek help without your parents knowing)? (12.8) |  |  |  |
| 1. Were you given enough explanations for test results (e.g., blood tests, questionnaires)? (8.3) |  |  |  |
| 1. Were you given enough information about your current treatment(s) (e.g., what they are for, possible side-effects, performing self-injections)? (11.7) |  |  |  |
| 1. Were you given enough information about your condition and how to prevent and/or take care of a health problem by yourself? (6) |  |  |  |
| 1. Were you involved in making decisions about your future healthcare plans, such as trying out different treatment options? (11.7) |  |  |  |
| 1. Were you given enough information about other services and how you can be referred to them (e.g., seeing a physiotherapist or psychologist)? (3.7) |  |  |  |
| 1. Were you given enough information about organisations that can provide support, such as the opportunity to meet other young people with similar conditions? (7.1) |  |  |  |
