# Supplementary material for: Development of a benchmarking toolkit for adolescent and young adult rheumatology services (BeTAR)
Source: Pediatr Rheumatol Online J. 2019 May 21;17:23. doi: 10.1186/s12969-019-0323-8 (PMC6528251; doi:10.1186/s12969-019-0323-8)
Supplement: Supplementary file 5 — HCP toolkit. (DOCX 109 kb) [file 12969_2019_323_MOESM5_ESM.docx]

**Additional file 5:** HCP toolkit

Category A: Holistic assessments & treatments

**A1** A y**oung person with an inflammatory disease at your centre has access to** which of the following healthcare professionals? *(select as many options as applies)*
 
Please also provide details of the average waiting time for the first appointment with the healthcare professional once a referral is made by a rheumatologist.

**Score = 3.1**

|  | Yes--average waiting time < 6 weeks | Yes--average waiting time > 6 weeks | Yes--not sure about waiting time | No access to this service |
| --- | --- | --- | --- | --- |
| physiotherapist |  |  |  |  |
| nurse specialist |  |  |  |  |
| ophthalmologist |  |  |  |  |
| pain management centre |  |  |  |  |
| occupational therapist |  |  |  |  |
| clinical psychologist |  |  |  |  |
| podiatrist/orthoptist |  |  |  |  |
| social worker |  |  |  |  |
| youth worker |  |  |  |  |
| prescribing pharmacist allied to the rheumatology team |  |  |  |  |
| hand therapist |  |  |  |  |

**Score = 5.7**

**A2** Do you have any challenges in meeting your own centre’s agreed or nationally published guidance for starting and monitoring anti-inflammatory treatment? *(select as many options as applies)*

- Blood monitoring of anti-inflammatory treatment--Please specify which guidelines you are using (e.g., BSPAR or local guideline) ________________________________________________
- Setting up shared care and communicating with local general practitioner/primary care/community nurses
- Urgent appointments with local care/network team
- adequate administrative support
- Other challenges—please specify ________________________________________________
- ⊗No challenges

**A3** In addition to peer learning in direct clinical practice, what additional training does your unit **offer for managing juvenile onset rheumatic diseases**: *(select as many options as applies)*

**Score = 6.2**

|  | Available for members of the medical team | Available for members of the multidisciplinary team | Not available |
| --- | --- | --- | --- |
| Dedicated face-to-face teaching sessions/seminars for wider rheumatology curriculum |  |  |  |
| Online learning resources/training or e-learning (e.g., from EULAR) * |  |  |  |
| Attendance at national/regional meetings |  |  |  |
| Textbooks & medical journals |  |  |  |
| Other—please specify (if none, tick "not available") |  |  |  |

* Your own or signposted to external online resources/training that is easily accessible for healthcare professionals (e.g., from the trust's intranet or shared drive)

**A4** 4We are interested in establishing if your unit provides specific training in core adolescent healthcare skills ( e.g., communication skills, developmental physiology). In addition to peer learning in direct clinical practice, what additional **adolescent health care skills training** does your unit provide: *(select as many options as applies)*

**Score = 3.4**

|  | Available for members of the medical team | Available for members of the multidisciplinary team | Not available |
| --- | --- | --- | --- |
| Dedicated face-to-face teaching sessions/seminars for wider rheumatology curriculum |  |  |  |
| Online resources/training or e-learning |  |  |  |
| Attendance at national/regional meetings |  |  |  |
| Textbooks & medical journals |  |  |  |
| 4Other—please specify (if none tick "not available") |  |  |  |

**A5** Feedback from young people suggested that pain and psychosocial assessments are important. Which of the following outcomes do you assess routinely at each consultation using validated, developmentally appropriate measures, **and make adjustments to treatments if necessary**? *(select as many options as applies)*

**Score = 2.1**

- Pain assessment--Please specify what outcome measure/tool you use ________________________________________________
- Functional health (i.e., ability to perform normal daily activities)--Please specify what outcome measures/tools you use ________________________________________________
- Standardised disease activity measure--Please specify what outcome measures/tools you use ________________________________________________
- Psychosocial health screening--Please specify what outcome measures/tools you use ________________________________________________
- ⊗None

**Score = 2.3**

**A6** Does your centre provide access to biologic therapies?

- Yes
- No

**A7** **On average**, young people can start anti-inflammatory treatment how long after their first visit?

**Score = 3**

- < 2 weeks
- 2-4 weeks
- > 4 weeks

**A8** Do you provide access to joint injections?

**Score = 2.3**

- Yes
- No

Display This Question:

If Do you provide access to joint injections? = Yes

**Score = 3**

**A9** **On average**, how long do young people wait for joint injections to be performed once a decision is made?

- On the same day
- 1 day to 1 week
- 1 to 4 weeks
- > 4 weeks

Display This Question:

If Do you provide access to joint injections? = Yes

**Score = 1.4**

**A10** Please select which of the following young people have easy/routine access to during joint injections: *(select as many options as applies)*

- Entonox
- General anaesthetics
- Local anaesthetics
- ⊗None

| Page Break |  |
| --- | --- |

-
Category B: Providing Information & Involvement

Display This Question:

If If Please provide the age range of patients that your centre provides care for: Minimum age Is Less Than 16

**Score = 2.2**

**B1** Do you copy clinic letters directly to the patient as part of your routine clinical practice?

- No, letters are only addressed to parents
- Only when young people are 16 years old or older
- Yes if developmentally appropriate even if young person is less than 16 years old
- ⊗Only when patients/parents ask for it

Display This Question:

If If Please provide the age range of patients that your centre provides care for: Minimum age Is Greater Than 15

**B1** Do you copy clinic letters directly to the patient as part of your routine clinical practice?

**Score = 2.2**

- Yes, letters are addressed directly to the patient
- ⊗Only when patients ask for it

**B2** Feedback from young people suggested that it is important for them to receive summaries and explanations of their assessment results. Do you routinely share and explain the following assessment results with young people as part of routine clinical practice (e.g., included in clinic letters)?*(select as many options as applies)*

**Score = 4.4**

|  | Share summaries of the result | Provide explanations of what the results mean | We don't share this routinely/shared only upon request |
| --- | --- | --- | --- |
| Blood test results |  |  |  |
| Questionnaire results (e.g., health assessment questionnaire) |  |  |  |

**Score = 2.2**

**B3** How long, on average, does it take for your unit to send out clinic letters?

- < 1 week
- 1-2 weeks
- > 2 weeks

**Score = 1**

**B4** Do you have a rheumatology nurse, with expertise in treating young people rheumatology, who can teach self-injections and administration in an age-appropriate way?

- Yes
- No

**B5a** Young people would like **both** print (e.g., leaflet) and electronic (e.g., your hospital's website, signposts to external websites, downloadable files) information and resources. Please indicate if you provide developmentally appropriate information/resources on the following topics *(select as many options as applies)*:

**Score = 4.3**

|  | Print | Electronic | Not provided |
| --- | --- | --- | --- |
| Young people's rheumatic condition--Please specify what type of information/resources you provide |  |  |  |
| Medications and side effects--Please specify what type of information/resources you provide |  |  |  |
| Pain management techniques--Please specify what type of information/resources you provide |  |  |  |
| Social/peer support--Please specify what type of information/resources you provide |  |  |  |
| Stress & emotional wellbeing--Please specify what type of information/resources you provide |  |  |  |
| Employment/vocational issues--Please specify what type of information/resources you provide |  |  |  |
| Educational issues--Please specify what type of information/resources you provide |  |  |  |
| Self-management/resilience--Please specify what type of information/resources you provide |  |  |  |

**Score = 0**

**B5b** Do you provide your own information/resources on these topics or use/signpost to information from other organisations/charities

- Own resources
- External resources--please specify ________________________________________________

**Score = 0.6**

**B5c** How often do you update these information/resources?

- Annually
- Every 1-5 years
- Not updated in the last 5 years

Please indicate if you are a: = Rheumatologist Clinical Nurse Specialist

- Don't know

Display This Question:

If If Please provide the age range of patients that your centre provides care for: Minimum age Is Less Than 16

**Score = 0.5**

**B5d** Are these resources primarily written for parents or the young person?

- Primarily for parents
- Primarily for the young person
- There are different versions for both parents and young people

**Score = 1.8**

**B6** What support do you provide for young people's school/work place?*(select as many options as applies)*

- Written information (or signpost to websites with) appropriate written educational information regarding young people's specific rheumatic condition for teachers/employers--Please specify what type of information/resources you provide ________________________________________________
- Have a member of rheumatology team who can liaise with young people's school/university/work place
- ⊗None of the above

**Score = 4.4**

**B7** Feedback from young people suggested that shared decision-making in all aspects of their treatment and care is important—does your unit have a routine way of documenting shared decision making? *(select as many options as applies)*

- Yes, for routine clinical practice--please specify how it's documented ________________________________________________
- Yes, for the transition process--please specify how it's documented ________________________________________________
- ⊗No

**Score = 0.3**

**B8** Are young people informed about and given the chance to participate in research projects **on a regular basis**?

- Yes
- No

**B9** Do you involve young people (at least annually) in the design, delivery, and evaluation of services, and**demonstrate how patient feedback is used to inform service delivery?**

**Score = 0.3**

- Yes--please specify how this is done ________________________________________________
- No

| Page Break |  |
| --- | --- |

-
Category C: Service Accessibility & Environment​

**C1a** Feedback from young people suggested that it is important for them to see a member of the rheumatology medical team once a year **WHEN THEIR DISEASE IS STABLE**. Is this standard practice at your unit?

**Score = 1.6**

- Yes
- No--please provide detail of frequency of followup ________________________________________________

**C1b** Feedback from young people suggested that **WHEN THEIR DISEASE IS NOT STABLE,**it is important for them to see a member of the rheumatology medical team at least once every 3-6 months until complete remission/low disease activity. Is this standard practice at your unit?

**Score = 2.5**

- Yes
- No--please provide detail of frequency of followup ________________________________________________

**C2** How does your unit manage urgent consultations?*(select as many options as applies)*

**Score = 4.6**

- 2Ring-fenced regular appointments--please provide details of frequency ________________________________________________
- 2Emergency ad-hoc appointments--please specify a time-frame for these appointments ________________________________________________
- 2Emergency phone consultation--Please specify a time-frame for these consultations ________________________________________________
- Other--please specify ________________________________________________
- ⊗No urgent appointments available

**Score = 1.4**

**C3a** How can young people contact the service with questions about their care and about accessing routine or urgent appointments?*(select as many options as applies)*

- Emails
- Text messages
- 3Phone calls
- Mobile apps
- ⊗None of the above

**C3b** Feedback from young people suggested that it can be difficult to reach multidisciplinary services who are part of the rheumatology medical/nursing team. Please indicate how young people can contact multidisciplinary services with questions about their referrals, appointments, and to discuss any problems: *(select as many options as applies)*

**Score = 0.5**

- 1Emails
- 1Text messages
- 1Phone calls
- Mobile apps
- Other--please specify ________________________________________________
- ⊗None of the above

Display This Question:

If How can young people contact the service with questions about their care and about accessing rout... != None of the above

Or Feedback from young people suggested that it can be difficult to reach multidisciplinary services... != None of the above

**Score = 1.2**

**C4** On average, how long do patients/families have to wait for their questions to be answered?

- 12less than or equal to 1 working day
- 2 to 3 working days
- ⊗more than 3 working days

**C5** Can young people request to consult/see a different rheumatologist at the centre (e.g., different gender)?

**Score = 3.6**

- Yes
- No

**Score = 4.7**

**C6a** Can young people see the same rheumatologist at each consultation?

- Yes--if requested
- Yes--they are always guaranteed to see the same Doctor at each appointment
- No

Display This Question:

If Can young people see the same rheumatologist at each consultation?  != Yes--they are always guaranteed to see the same Doctor at each appointment

**C6b** Feedback from young people suggested that it is important for their doctors to be familiar with their medical history. If there are a number of medical professionals working at your team and young people are not guaranteed to see the same doctor at each appointment, then do you have unified medical notes and direct handovers of previous care?

**Score = 1.7**

- Yes--please specify how this is documented ________________________________________________
- No

Display This Question:

If Can young people see the same rheumatologist at each consultation?  = No

**C6c** Feedback from young people suggested that it is important for them to be informed beforehand (e.g., in their clinic confirmation letter) which doctor they will see—is this standard practice at your unit?

**Score = 1**

- Yes
- No

**C7** Do you provide written information regarding confidentiality and privacy, and make sure that young people know they can seek help without parents’ knowledge/consent?

**Score = 2.6**

- Yes--Please specify what type of information/resources you provide ________________________________________________
- No

**Score = 2.6**

**C8** Are consultations given in a private area, and are other people present (e.g., trainees, researchers) only with young people's permission?

- Yes
- No

**Score = 1.3**

**C9** Do you have a dedicated:

|  | Yes | No |
| --- | --- | --- |
| adolescent/young person clinic? |  |  |
| adolescent/young person waiting room? |  |  |

**C10** Regarding clinic appointments/consultations, do you:

**Score = 0.6**

|  | Yes | No |
| --- | --- | --- |
| facilitate non face-to-face (e.g., telephone, skype) clinical consultations where appropriate? |  |  |
| arrange combined visits with other members of the multidisciplinary team? |  |  |
| offer out-of-working/school hour appointments that suit local needs (e.g., late afternoons after 17:00 or weekends)? |  |  |

**Score = 2**

**C11** For rheumatology consultations, do you provide:

|  | Yes | No--please specify length of appointment in text box |
| --- | --- | --- |
| at least 40 min. for new appointments? If no, please specify length of appointment in text box |  |  |
| at least 20 min. for follow-up rheumatology consultations? If no, please specify length of appointment in text box |  |  |

**Score = 1.8**

**C12** Can young people request longer appointments if needed?

- Yes
- No

Display This Question:

If If Please provide the age range of patients that your centre provides care for: Minimum age Is Less Than 16

And Please provide the age range of patients that your centre provides care for: Maximum age Is Greater Than 15

**Score = 0.5**

**C13** Do you routinely offer appointment reminders?*(select as many options as applies)*

|  | Reminder via post | Reminder via email | Reminder via text | Not offered |
| --- | --- | --- | --- | --- |
| Reminders for young people when they are 16 years old or older |  |  |  |  |
| Reminders for young people < 16 years old if developmentally appropriate |  |  |  |  |
| Reminders for parents |  |  |  |  |

Display This Question:

If If Please provide the age range of patients that your centre provides care for: Maximum age Is Less Than 16

**C13** Do you routinely offer appointment reminders?*(select as many options as applies)*

**Score = 0.5**

|  | Reminder via post | Reminder via email | Reminder via text | Not offered |
| --- | --- | --- | --- | --- |
| Reminders for young people if developmentally appropriate |  |  |  |  |
| Reminders for parents |  |  |  |  |

Display This Question:

If If Please provide the age range of patients that your centre provides care for: Minimum age Is Greater Than 15

**Score = 0.5**

**C13** Do you routinely offer appointment reminders for young people?*(select as many options as applies)*

- Reminder via post
- Reminder via email
- Reminder via text
- ⊗Not offered

**Score = 1.4**

**C14a** Please specify the number of non-attendance before discharging young people from your service **FOR NEW PATIENTS**:

|  | After 1 non-attendance | After 2 non-attendances | After 3 non-attendances | After more than 3 non-attendances |
| --- | --- | --- | --- | --- |
| Patients who are stable and off medications--please specify how you offer re-arrangements after non-attendance |  |  |  |  |
| Patients who are receiving treatment(s)--please specify how you offer re-arrangements after non-attendance |  |  |  |  |

**C14b** Please specify the number of non-attendance before discharging young people from your service **FOR FOLLOW-UP PATIENTS**:

**Score = 1.4**

|  | After 1 non-attendance | After 2 non-attendances | After 3 non-attendances | After more than 3 non-attendances |
| --- | --- | --- | --- | --- |
| Patients who are stable and off medications--please specify how you offer re-arrangements after non-attendance |  |  |  |  |
| Patients who are receiving treatment(s)--please specify how you offer re-arrangements after non-attendance |  |  |  |  |

**C15** Do you explain your non-attendance/discharge policy?*(select as many options as applies)*

**Score = 1.6**

|  | post | emails | phone | text | At clinic consultations | No explanation/pre-warning |
| --- | --- | --- | --- | --- | --- | --- |
| Explained to young people by... |  |  |  |  |  |  |
| If Please provide the age range of patients that your centre provides care for: Minimum age Is Less Than 16  Explained to parents by... |  |  |  |  |  |  |

| Page Break |  |
| --- | --- |

-
Category D: Continuity of Care

**D1** Is your centre part of a formal transition pathway with a local partner (e.g., do you have an agreed link with local paediatric or adult centre in terms of transitioning young people from, or to, your service)?

**Score = 1.7**

- No
- Yes--this is NOT documented in a written policy/protocol
- Yes--this is documented in a written policy/protocol and updated annually
- Yes--this is documented in a written policy/protocol and updated every 1-5 years
- Yes--this is documented in a written policy/protocol, but was not updated in the last 5 years

Please indicate if you are a: = Rheumatologist Clinical Nurse Specialist

- Yes, but not sure if it's documented or how often it is updated

**D2** Alongside medical transition, young people also want coordinated transitions of their multidisciplinary care. Is there a specific multidisciplinary transition pathway that is agreed with all multidisciplinary team members involved in young people's care?

**Score = 1.7**

- No
- Yes--this is NOT documented in a written policy/protocol
- Yes--this is documented in a written policy/protocol and updated annually
- Yes--this is documented in a written policy/protocol and updated every 1-5 years
- Yes--this is documented in a written policy/protocol, but was not updated in the last 5 years

Please indicate if you are a: = Rheumatologist Clinical Nurse Specialist

- Yes, but not sure if it's documented or how often it is updated

Display This Question:

If If Please provide the age range of patients that your centre provides care for: Minimum age Is Less Than 16

**Score = 2.1**

**D3** When preparing young people for transition, do you:

- Start the transition process in a developmentally appropriate way around 11-14 years old?
- Provide (or signpost to websites with) appropriate written information and advice on transition and what to expect for young people and parents? -- Please specify what type of information/resources you provide ________________________________________________
- Work together with young people to develop an individual transition plan that is documented in writing and shared with young people and parents? -- please specify how it's documented ________________________________________________
- ⊗None of the above

Display This Question:

If If Please provide the age range of patients that your centre provides care for: Minimum age Is Greater Than 15

**Score = 2.1**

**D3** Do you always have adequate transfer information from the paediatric service?

- 1Yes--please specify ________________________________________________
- No--please specify ________________________________________________

**Score = 0.7**

**D4** Do you allow flexibility for the age of transition, depending on young people's developmental maturity and readiness?

- Yes
- No

**D5** When young people transition from or into your care, do you have*(select as many options as applies)*:

**Score = 1.8**

|  | Yes--this policy is updated annually | Yes--this policy is updated every 1-5 years | Yes--this policy has not been updated in the last 5 years | Please indicate if you are a: = Rheumatologist Clinical Nurse Specialist  Yes--but not clear when this policy is updated | No |
| --- | --- | --- | --- | --- | --- |
| an agreed written policy to confirm successful transfer (e.g., 2 attendances in the adult centre as per NICE guidelines)?--please specify what your agreed mechanisms/policies are |  |  |  |  |  |
| an agreed written policy to chase young people and 'safety net' their care if they fail to attend clinics post transfer?--please specify what your agreed mechanisms/policies are |  |  |  |  |  |

**D6** Do you have a key individual to help with coordinating care, discussing transitional plans, and explaining different models of care at different centres (e.g., that they will be seen by a team of different consultants, not necessarily an individual consultant)?

**Score = 0.8**

- Yes--please specify who this is (e.g., nurse, consultant, support worker) ________________________________________________
- No

**D7** Will the young person be able to meet at least one staff member (who is familiar with the transition process) whom they can see **before** the transition (e.g., at the paediatric hospital) **AND after** the transition (e.g., at the adult hospital)?

**Score = 0.8**

- Yes--please specify who this is (e.g., nurse, consultant, support worker) ________________________________________________
- No

| Page Break |  |
| --- | --- |
